# The surged faradic stimulation to the pelvic floor muscles as an adjunct to the medical management in children with rectal prolapse

**DOI:** 10.1186/1471-2431-9-44

**Published:** 2009-07-14

**Authors:** Simmi K Ratan, Kamal Nain Rattan, Poonam Jhajhria, Yogesh Parshad Mathur, Atul Jhanwar, Dimple Kondal

**Affiliations:** 1 Department of Pediatric Surgery, Post Graduate Institute of Medical Sciences, Rohtak, Haryana, India; 2 Department of Physiotherapy, Post Graduate Institute of Medical Sciences, Rohtak, Haryana, India; 3 Department of Biostatistics, All India Institute of Medical Sciences, New Delhi, India

## Abstract

**Background:**

To assess the role of the surged faradic stimulation to the pelvic floor muscles as an adjunct to the conservative management in the children of idiopathic rectal prolapse

**Methods:**

*Study design*: Prospective

*Setting*: Pediatric Surgery Department, Pt BD Sharma, Post Graduate Institute of Medical Sciences, Rohtak

*Subjects*: 47 consecutive children with idiopathic rectal prolapse attending the Pediatric Surgery out patient department from July 2005 to June 2006

*Methodology*: The information pertaining to duration and the extent of rectal prolapse, predisposing or associated medical conditions, results of local clinical examination were noted. Surged faradic stimulation using modified intraluminal rectal probe, was given on the alternate days. The conventional conservative medical management was also continued. The extent of relief and the number of the sittings of faradic stimulation required were noted at various stages of follow-ups

*Statistical Methods*: Mean values between those completely cured and others; poor responders and others were compared with t-test and proportions were compared with Chi square test. The p-value < 0.05 was considered statistically significant.

**Results:**

The mean number of sittings in the completely cured group (n = **28**(64%)) was (12.4 ± 7.8) and was comparable with very poor responder (n = 6(13%). There was higher percentage of relief (76%) at the first follow up (at 15 days) in completely cured Vs other (37%) and also the poor responders showed (20%) Vs other (68%) and was statistically significant.

**Conclusion:**

With use of faradic stimulation, even the long-standing rectal prolapse can be fully cured. The follow up visit at 2 weeks is very important to gauge the likely success of this modality in treatment of the patients with rectal prolapse. Those showing poor response at this stage may require alternative treatment or take a long time to get cured

## Background

Rectal prolapse is a common condition in children in the underdeveloped nations, albeit with unclear etiology. The commonest predisposing factors are the diseases leading to malnutrition (e.g. amebiasis, giardiasis, worms) leading to disappearance of the ischio-rectal fat and causing lack of rectal support. A persistently increased intra -abdominal pressure e.g. due to constipation, frequent cough, has also been implicated. Among the anatomical factors that had to the development of rectal prolapse in children are the vertical course of rectum, a low-position of rectum in relation to other pelvic organs and lack of levator support [1,2]. There are other authors who believe that the rectal prolapse in children mainly involves the mucosa; and the muscle coats are either not involved or are involved at a later stage [3]. This reason is forwarded to explain the noted higher frequency of rectal prolapse in infants [4]. It is, therefore, on this ground that the prolapse in children is supposedly different from the adults (as in adults the weakness of the pelvic floor is the predominant etiological factor) [5].

Based on these principles, the conservative treatment has remained the initial cornerstone of the rectal prolapse management in children and the interventional therapies (injection treatment and surgical operations) have been reserved for the non-responders [2,4]. By and large, the conservative treatment includes use of stools softeners, strapping of the buttocks to prevent recurrence of prolapse and defecation in lateral position [2].

In the present study, we added one more component to the prevalent conservative treatment and that is, the use of surged faradic current for stimulating external anal sphincter and the pubo-rectalis sling. This was contemplated seeing the fair results of using this current in our patients where the fecal incontinence had resulted due to poorly developed or neurologically weak pelvic floor muscles. Similar observations had been earlier reported by various workers also [6-8]. The surged faradic stimulus was given with the help of rectal probe that was designed so as stimulate the external anal sphincter and the weak pelvic floor muscles (when introduced passed intraluminally). The rationale of the therapy is that the strengthened external anal sphincter and pelvic floor muscles would support the rectum adequately causing amelioration of the rectal prolapse. Though initially employed in the children presenting with failed conservative management, the therapy was subsequently added as adjunct to the conservative management for every case with rectal prolapse, so as to expedite the relief. To the best of our knowledge, no similar study has been reported in the English literature till date.

## Methods

Forty seven consecutive children with symptomatic rectal prolapse from July 2005 to June 2006 were included in the study. A detailed history was obtained from the parents and was adjudged to be reliable for all patients. Thereafter they were examined by the members of pediatric surgical team having more than 10 years of experience in specialty of Pediatric Surgery. Most study subjects had long standing prolapse and were awaiting their turn for the definitive surgical treatment while the surged faradic stimulation as was added as an adjunct to conservative management. The data was collected on their sex, age, duration and extent of prolapse (length and the coats of rectum involved), anal tone, the preceding illness, if any, family history, duration of conservative management and the extent of relief obtained thereby. Prior conservative management was said to have been given if the child had taken this treatment at a stretch at least for four weeks. All the early presenters (n = 12) in the study (those presenting within 2 weeks of initiation of symptoms), that is, with conservative management of lesser than 4 weeks, were considered to have taken no prior conservative management. The extent of prolapse was adjudged mainly on the subjective assessment of the reporting parent. However, an objective assessment was also done by the physiotherapist while child was being subjected to faradic stimulation. It was rated as complete if all rectal coats could be visualized through the entire circumference of the prolapsed segment. The assessment of anal tone was done subjectively at the initial examination by experienced pediatric surgeons. Only gross deviation from normal could be picked up in this manner in the outpatient setting. as invariably the children were apprehensive and tending to contract the entire pelvic floor musculature in an attempt to resist anal examination. The children were given surged faradic stimuli to the external anal sphincter and the pelvic floor muscles while continuing with the conservative management (stool softeners, high protein high caloric diet). A good reassurance and a mild sedative for more apprehensive child were enough to derive co-operation from them. The older children (5 years or more) were taught the abdominal and pelvic floor muscle exercises; whereas, for the younger children, the mothers were taught the passive maneuvers to achieve the same goal. They subjects and their parents were instructed to strictly adhere to this regime of treatment or face failure or relapse. No fixed period for this adjunct treatment was contemplated and the final response was adjudged as the one present at the 'exit point' of the patient from the study.

### The procedure

**Figure 1:**
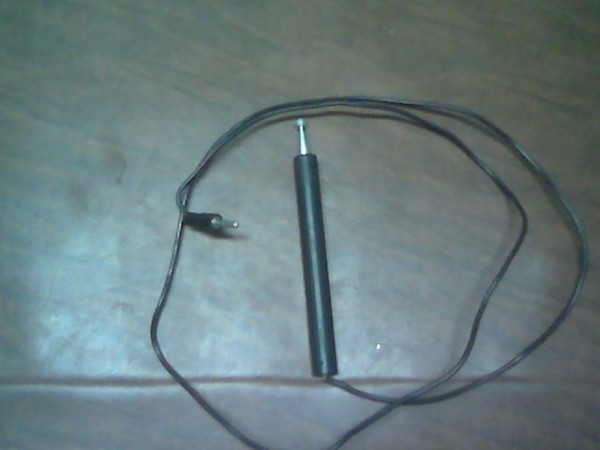
**Intrarectal pen electrode used for giving faradic stimulation**.

**Figure 2:**
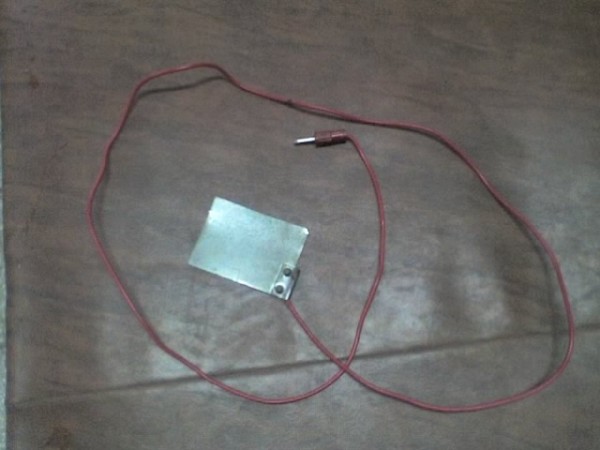
**The inactive electrode**.

The child was made to lie in prone position. Electrical stimulation was given using Timpac muscle stimulator TMS-10 with intrarectal pen electrode (our modification) [Fig 1], while inactive electrode [fig 2] was placed under patient's pelvis The electrical stimulation was achieved with pulses of duration of 1 ms with a frequency of 30 Hz(faradic type pulses)

Inactive electrode(large pad electrode) was placed under lower abdomen. Active electrode (Pen electrode) was used for the external anal sphincteric stimulation and for pelvic floor muscle stimulation. The electrode was inserted for over a short distance into the rectum(by1/4 inch or so). Intensity of the current used was variable with each patient. It was gradually increased till observable muscle contraction was observed i.e. anal wink. The patient was asked to do voluntary anal contraction with each stimulus. About 90 contractions were given in each session to avoid muscle fatigue, over 3 minutes (30 contractions per minute). Each patient was given1 session per day 3 times a week (on alternate days). Meanwhile the patient was asked to do pelvic floor exercises; mainly the tightening of the abdominal muscles, the thigh adductors and gluteals and drawing up the pelvic floor muscles for 5 sec and then rest. Other exercises included strengthening of spinal extensors and abdominal muscles. The patients were asked to do them as frequently as possible with a minimum of twice daily.

The relief was categorized as 50%, 75% or 100% depending both on the reduction in the size of the prolapsed rectum and the frequency of prolapse (spontaneous or while straining; everytime with straining or occasional), as deduced from the combined assessment done from information delivered by the parents and the examination findings of the physiotherapists The initial response at first follow up at 2 weeks of therapy was also noted. The response was gauged with respect to the average number of sittings undertaken for getting 100% cure of rectal prolapse. The first instance when the parents reported the prolapse not to have appeared even after straining was marked. It was considered as the 'end point' if the prolapse did not appear for two consecutive weeks. Any appearance of the prolapse after 2 weeks after its disappearance qualified for a 'relapse'. Thereafter, follow up visits were advised at 4, 8 and 12 week after stopping of treatment to confirm the complete amelioration. In those who failed to turn up on the due date, the same was enquired telephonically. However, in subjects who exited the therapy short of complete relief, the degree of relief and the number of sittings at the exit point were taken for computation purpose. Any telephonic report of full relief of prolapse in these subjects was ignored for the want of substantiation of the same by the supervising clinicians or physiotherapists. For the non responders (50% or less) the cause of failure was noted.

### Statistical Methods

STATA version 9.0 was used. The responders and non responders were identified as two groups. The difference of mean for different parameters between the two groups was tested by 't' test and difference between the propotions by chi-square test. A p value of < 0.05 was taken as statistically significant.

## Results

The mean age of subjects was 4.5 year, ranging from 4 months to 12 years. The mean duration of symptoms being 9.3 month, ranging from 1 day to 4 years. There were 12 early presenters. The extent of prolapse was partial, that is, not involving the all coats and circumference of rectum, in 40% (n = 19) and total in 60% (n = 28). The prior history of illness present were as: bleeding (n = 4) diarrhea (n = 14), constipation (n = 14), other illness (n = 9). The family history was positive in 9% patients (n = 4) only. The prior conservative management was taken by about 70% children (n = 35) and only 32% (n = 15) of them had shown partial response to this treatment. Most children (n = 44, 94%) had average build. A decreased anal tone was noted among 9 children (19%) only. The extent of prolapse was less than 3 cm in 64% (n = 30) and the in others it was longer than 3 cm. The symptomatic relief for associated symptoms as bleeding and pain, was quite quick with above stated regime, with all subjects getting relieved of the associated symptoms by the first follow up at 2 weeks. The children tolerated this therapy regime quite well, though towards the beginning of therapy they were apprehensive and reluctant. A painless therapy with quick amelioration of their symptoms proved to be morale boosters.

Twenty eight children responded fully; while 19 showed partial incomplete response. There were 10 children who had < 50% relief. Of them 4 had taken only 6 sittings of faradic stimuation (only 2 weeks of therapy). Only three patient had taken faradic stimulation for beyond 4 weeks and still got relieved lesser than 50% relief. Of them, two had suffered the illness for 2, 6 months respectively while the third subject had a shorter duration (10 days) The maximum number of sitting to reach 100% relief was 36; while the minimum number was 1, surprisingly. The tables showing the distribution of 'extent of relief' with respect to 'duration of the presenting symptoms' (Table 1) and the 'number of faradic stimulation sittings' (Table 2) are given. Briefly, they show a better response for subjects who had long standing rectal prolapse and were therapy-compliant. In fact, 6 of 9 patients with 75% relief on therapy affirmed to have been totally cured on telephonic enquiry 12 weeks after the exit point from therapy, but the same could not be used for computation of results in the present study in absence of substantiation of the same by the supervising clinicians. No patient reported recurrence of symptoms after achieving complete relief.

**Table 1:** The relation between the duration of rectal prolapse and the extent of relief

**Duration of symp**	**<= 50% relief (n = 10)**	**Upto 75%relief** **(n = 9)**	**100% relief** **(n = 28)**
<= 4 wk(n = 12)	2	3	7

>4 to <= 12 wk (n = 10)	2	1	7

>12 wk to <= 1 yr (n = 15)	5	3	7

>1 yr (n = 10)	1	2	7

**Table 2:** The relation between the number of faradic stimulation sittings taken and the extent of relief

**No of faradic stimultn sittings**	<= 50% relief (n = 10) *	**Upto 75%relief** **(n = 9)**	**100% relief** **(n = 28)**
6 (for 2 wks) (n = 11)	4	2	5

> 6–18 (upto 6 wk) (n = 29)	5	6	18

> 18–36(upto12 wk) (n = 7)	1	1	5

• Seven of these patients were non-compliant

• In a week three sittings were given on alternate days

The mean number of sitting in these fully cured (12.4 ± 7.8) did not differ much from those with poor response i.e. <50% cure (11.75 ± 6.9). Also, no statistically significant difference was noted between the number sittings showing < 50% response (6.7 ± 4.1) and those showing >50% response (8.4 ± 6.0). However, the former showed a higher percentage of relief at the first follow up visit (76% ± 20%) in contrast to (20 ± 24%) for the non responders at 2 weeks of beginning of the therapy. This difference was found to be significant (p = 0.01). However, as expected all non-compliance emerged out to be the strongest predictor of non-response to this treatment (p = 0.001).

## Discussion

The surged faradic current has found wide application to improving the strength and vascularity of various groups of muscles including external anal sphincter [9]. In the present study, besides stimulating and strengthening the external anal sphincter, an improvision was done to stimulate the pelvic floor muscle as well. This was to take care of the causative factor of poor pelvic floor muscle support in rectal prolapse in children. As we noted that most children presented to us had average health, long duration of rectal prolapse of 9.3 month (average) that had involved all coats of rectum. Therefore, our study supports time conclusion of Fowler that the common form of rectal prolapse involve all coats of rectum [10]. However, another reason could be that the initial mucosal prolapse, over the time, involved all the layers of rectum as only 17% (n = 2 out of 12) of those early presenters (within days) had total rectal prolapse. On a wider perspective, it can be deduced that the chronically present rectal prolapse leads to weakness/atony of the pelvic floor muscles that required to be improved by their stimulation and/or exercise. Hence, inclusion of faradic stimulation of the pelvic floor muscles should form an integral part of the conservative therapy for the rectal prolapse.

We could cure 28 (64%) children fully using this strategy; of which 21 (70%) whom had received the conventional conservative management for rectal prolapse for longer than 3 months and would have required some form of interventional therapy. This shows that the judicious application of a simple non-invasive technique of faradic stimulation can cure long standing rectal prolapse and obviates any need of any other form of intervention. As expected, the cure rate was poor in the non complaint children. On the other hand, results were gratifying in those with long standing prolapse of over 1 year duration (Table 1). These subjects were found to be most compliant for this therapy as well. Perhaps chronic suffering and a failure to respond to the conventional conservative management acted as a motivational force for compliance to the regime of pelvic floor muscle stimulation/exercise in these children. The other factor for a comparatively lesser degree of response in those with shorter duration of rectal prolapse could be a lesser role of pelvic floor weakness in these subjects.

It is possible that this therapy alone could have resulted in total cure in all our patients had they all of them remained compliant to the therapy or the therapy would have continued for a fairly long period. This is because additional subjects with lesser than 'complete relief' affirmed to have achieved complete response after 12 weeks of follow up enquiry, as has been given under 'results' section. Still, we believe that 12 week is an optimal period after which most parents were found to lose patience and begged for the alternate treatment. Besides, we also found that the pattern of response of the patient at 2 week of faradic treatment (i.e. 6 – 8 sittings) have great bearing in prediction of success of this treatment. Those showing >50% response at this stage just need to be compliant and relief generally follows over next few weeks. However, all the poor responders at this stage in our study did not reach their final goal within our period of observation. The reason for this response may be related to the variable role of the weakness of the pelvic floor muscles in the genesis of rectal prolapse in different children.

This modality can be criticized for the prolonged duration of treatment and the need for the frequent hospital visits causing inconvenience to the patient and the parents. However, when compared with the inconvenience of undergoing general anaesthesia for the surgical procedure (even repeated general anaesthesia as are required for the injection therapy, i.e. upto 2–3 times) and likelihood of their complications (e.g. infection, abcesses for injection treatment) [1,3,11], this inconvenience may appear quite trivial. Above all, this modality of treatment is not marred by any side effect; except that in few cases the total cure may not follow early or at all. However, the trend of response can be predicted fairly early during the course of the treatment. We are presently considering the non-responders of our study for Thiersch wiring, out of many procedures advocated for rectal prolapse, as it is easy to perform and has yielded optimum results in our hands in the past.

Recently, extracorporeal magnetic innervation (Ex MI) therapy has been tried to improve results in patients with incontinence. ExMI technology works by producing a highly focused, time-varying magnetic field that penetrates deep into the perineum, innervating the pelvic floor muscles by activating motor neurons. Pulses of steep gradient magnetic flux are produced by the therapy head. These fields penetrate the patient's perineum and initiate nerve impulses. Yokoyama et al have used this treatment modality in patients with urinary incontinence and found good results in those with stress incontinence [12]. In future this therapy may also be tried to increase the strength of the pelvic floor muscles in patients with rectal prolapse.

## Conclusion

Rectal prolapse is benign condition that causes great concern to the patient and the parents. An adequate trial of conservative management, along with faradic stimulation of pelvic floor can totally cure most of these children, if proper heed is paid to the compliance for the treatment regime and to the pelvic floor and abdominal muscles exercises. Our study does point to the fact that pelvic floor weakness does significantly contribute in the pathogenesis/pathology of rectal prolapse in children as well.

## Competing interests

The authors declare that they have no competing interests.

## Authors' contributions

SKR conceived of the study and participated in its design and co-ordination, monitored patients' response and follow ups. KNR supervised study. AJ collected patient data. YPM and PJ gave faradic stimulation therapy and monitored the response. DK performed statistical analysis. All authors read and approved the final manuscript

## Pre-publication history

The pre-publication history for this paper can be accessed here:


